# LncRNA LOC730101 Promotes Darolutamide Resistance in Prostate Cancer by Suppressing miR-1-3p

**DOI:** 10.3390/cancers16142594

**Published:** 2024-07-20

**Authors:** Tianyi Zhou, Steven Nguyen, Jacky Wu, Bin He, Qin Feng

**Affiliations:** 1 Center for Nuclear Receptors and Cell Signaling, Department of Biology and Biochemistry, University of Houston, Houston, TX 77204, USA; 2 Immunobiology & Transplant Science Center, Houston Methodist Research Institute, Houston, TX 77030, USA; 3 Department of Medicine-Cancer Biology, Weill Cornell Medicine, Cornell University, New York, NY 10065, USA

**Keywords:** prostate cancer, antiandrogen, darolutamide, lncRNA, miRNA

## Abstract

**Simple Summary:**

This study investigates the role of non-coding RNAs in resistance to the antiandrogen drug darolutamide in prostate cancer. RNA sequencing identified LOC730101 as a significantly elevated lncRNA in darolutamide-resistant cancer cells. Silencing LOC730101 reduced cancer cell proliferation, in support of its role in drug-resistant cancer growth. Elevated LOC730101 levels were observed in metastatic castration-resistant prostate cancer (CRPC) tumors compared to primary prostate tumors. LOC730101 modulates the expression of cell cycle genes by suppressing microRNA miR-1-3p. Hi-C sequencing revealed that the *LOC730101* gene is situated within a dynamic topologically associating domain (TAD) that is activated in resistant cells. This study indicates that LOC730101 is crucial for darolutamide resistance and a potential target to overcome antiandrogen resistance in CRPC.

**Abstract:**

Antiandrogen is part of the standard-of-care treatment option for metastatic prostate cancer. However, prostate cancers frequently relapse, and the underlying resistance mechanism remains incompletely understood. This study seeks to investigate whether long non-coding RNAs (lncRNAs) contribute to the resistance against the latest antiandrogen drug, darolutamide. Our RNA sequencing analysis revealed significant overexpression of LOC730101 in darolutamide-resistant cancer cells compared to the parental cells. Elevated LOC730101 levels were also observed in clinical samples of metastatic castration-resistant prostate cancer (CRPC) compared to primary prostate cancer samples. Silencing LOC730101 with siRNA significantly impaired the growth of darolutamide-resistant cells. Additional RNA sequencing analysis identified a set of genes regulated by LOC730101, including key players in the cell cycle regulatory pathway. We further demonstrated that LOC730101 promotes darolutamide resistance by competitively inhibiting microRNA miR-1-3p. Moreover, by Hi-C sequencing, we found that *LOC730101* is located in a topologically associating domain (TAD) that undergoes specific gene induction in darolutamide-resistant cells. Collectively, our study demonstrates the crucial role of the lncRNA LOC730101 in darolutamide resistance and its potential as a target for overcoming antiandrogen resistance in CRPC.

## 1. Introduction

Darolutamide, marketed under the brand name Nubeqa, is the latest FDA-approved antiandrogen drug used to treat non-metastatic castration-resistant prostate cancer (CRPC) and metastatic hormone-sensitive prostate cancer [1,2]. Its mechanism of action involves binding to the androgen receptor (AR) with high affinity and effectively inhibiting AR signaling [3]. Darolutamide has been shown to inhibit both wild-type and mutant forms of AR, making it effective against prostate cancer cells that may have developed resistance to other forms of androgen deprivation therapy (ADT) [4].

The efficacy of Nubeqa was notably demonstrated in two clinical trials, ARAMIS for nonmetastatic CRPC and ARASENS for metastatic hormone-sensitive prostate cancer (mHSPC), where it was shown to delay disease progression, extend survival, and preserve the quality of life for patients [2,5,6]. Reflecting its growing acceptance and effectiveness, Nubeqa’s sales have substantially increased, nearly doubling in 2023. This trajectory supports Bayer’s forecast of reaching peak-year sales of USD 3 billion by 2030, indicating the potential of darolutamide in treating advanced prostate cancer [7].

Long non-coding RNAs (lncRNAs) are a distinct group of non-coding RNA transcripts that are 200 nucleotides or longer, without being translated into proteins. The human genome is estimated to contain over 58,000 lncRNA genes, accounting for around 68% of the human transcriptome [3]. LncRNAs play an important role in cancer development. Many lncRNAs have been identified to impact prostate cancer, with certain dysregulated lncRNAs finding applications in clinical settings as non-invasive biomarkers for early detection of prostate cancer, prognosis assessment, and monitoring of treatment efficacy [8]. For instance, a urine-based molecular assay that identifies the presence of the Prostate Cancer Antigen 3 (PCA3) lncRNA molecule is commonly used for prostate cancer diagnosis [9]. ARLNC1 (AR-regulated long non-coding RNA1) is a long non-coding RNA crucial for prostate cancer progression as it stabilizes AR transcription and enhances AR signaling, making it a novel therapeutic target [10].

LncRNAs can regulate gene expression at multiple levels, including transcriptional, post-transcriptional, and epigenetic mechanisms [11,12]. One of the important functions of lncRNA in regulating gene expression is to act as microRNA (miRNA) ‘sponges’. By binding and sequestering miRNAs, they effectively reduce their availability in the cell, thus inhibiting their ability to target mRNAs [13,14]. In addition, lncRNA can also directly bind to miRNAs and promote their degradation [15].

Despite the growing recognition of lncRNAs in cancer biology, research has predominantly focused on a limited number of these molecules. The study of lncRNAs in the context of therapeutic resistance, especially to newer treatments like darolutamide, remains largely unexplored. Therefore, our study aims to investigate whether lncRNAs contribute to the development of resistance against darolutamide in prostate cancer and to elucidate the molecular mechanisms involved. This could potentially identify new targets for overcoming drug resistance and improving prostate cancer treatment outcomes.

## 2. Materials and Methods

### 2.1. Cell Culture and Treatment

LNCaP, VCaP, and 293T cells were initially obtained from ATCC. DuCaP cells were obtained from the Pathology Shared Resource at the University of Colorado Cancer Center. LNCaP and DuCaP cells were cultured in RPMI 1640 medium supplemented with 10% fetal bovine serum (FBS) and 1% penicillin/streptomycin. To develop darolutamide-resistant LNCaP cells, we gradually increased darolutamide (TargetMol, Boston, MA, USA) concentrations to 10 μM. These cells were then continuously cultured in media containing 10 μM darolutamide for an additional 12 months. VCaP and 293T cells were cultured in DMEM supplemented with 10% FBS and 1% penicillin/streptomycin. All cell cultures were maintained in a humidified incubator with 5% CO_2_ at 37 °C. Mycoplasma contamination testing was conducted semiannually. For transient hormone treatments, Parental LNCaP cells were treated with DMSO (Vehicle), 10 nM R1881 (R1881), or 10 μM darolutamide (Daro) for 24 h.

### 2.2. RNA-Sequencing and Analysis

Total RNA was extracted using the RNeasy Plus Kit (Qiagen, Germantown, MD, USA). RNA quality control (QC) was assessed using a Bioanalyzer (Agilent, Santa Clara, CA, USA). RNA samples that met the quality criteria were sent to Novogene (Sacramento, CA, USA) for both library preparation and sequencing via the Illumina NextSeq550 platform. Raw FASTQ files were generated and subsequently filtered using the Trim-galore tool. The filtered FASTQ files were aligned using STAR 2.7, referencing the human genome hg19 (GRCh37) and the gencode.v19 comprehensive gene annotation file [16]. FeatureCounts was used to count gene fragments based on the gencode.v19 long non-coding RNA gene annotation file. Differential gene expression analysis was conducted with DESeq2, using the output from FeatureCounts as input [17]. Genes were considered significant if they had a *p*-value less than 0.05 and a base mean expression greater than 50. Data visualization, through volcano plots and heatmaps, was performed using ggplot2. A volcano plot was processed using EnhancedVolcano. Gene Set Enrichment Analysis (GSEA) was carried out using the GSEA desktop software v4.3.1. The R package ClusterProfiler v4.8.3 was used for enrichment pathway analysis [18]. Differentially expressed genes were analyzed using ClusterProfiler to evaluate their roles in biological processes, referencing both the GO_term and KEGG pathway databases.

### 2.3. siRNA and Transient Transfection

ON-TARGETplus SMARTpool siRNA (Horizon Discovery, Lafayette, CO, USA) was used for siRNA knockdown using the Lipofectamine RNAiMAX transfection reagent (Thermo Fisher Scientific, Waltham, MA, USA, #13778075) following the manufacturer’s instructions. Cells were transfected with either control siRNA or siLOC730101, with a final concentration of 20 nM. All other transient transfection experiments were performed using PEI-Max (Polysciences, Warrington, PA, USA).

### 2.4. Lentiviral Transduction

The miR-1-3p lentivirus-expressing plasmid vector was purchased from ABM (catalog #mh40026). It was transfected with VSVG and PAX2 (Addgene, Watertown, MA, USA) to produce lentivirus in 293T cells. The supernatant was collected and used to transduce the LNCaP, DuCaP, and VCaP prostate cancer cell lines. Three days after transduction, the transduced cells were selected with 5 µg/mL Puromycin for LNCaP and DuCaP cells or 2 µg/mL puromycin for VCaP cells.

### 2.5. Cell Viability Assay

Prostate cancer cells were seeded at a density of 1 × 10^5^ cells per well in flat-bottomed 96-well plates. A CellTiter-Glo^®^ Luminescent Cell Viability Assay (Promega, Madison, WI, USA) was used to measure cell viability every other day following the manufacturer’s instructions. Cell viability was determined by measuring the luminescence using the Synergy™ neo2 multi-mode reader (BioTek, Winooski, VT, USA).

### 2.6. Real-Time Quantitative PCR (RT-qPCR)

We used an RT-qPCR assay to determine the levels of gene expression. Prostate cancer cells were treated with different ligands for 24 h. Subsequent to this treatment, total RNAs were extracted using the RNeasy Plus Kit (Qiagen, #74034, USA) and quantified using the BioTek Synergy Neo2. An amount of 1 µg of total RNA from each sample was used for reverse transcription by the iScript cDNA Synthesis Kit (BioRad, Hercules, CA, USA). These cDNAs were used for qPCR in PerfeCTa qPCR Supermix (QuantaBio, Beverly, MA, USA, #95113-012) on the Thermo ABI 7500 Fast Real-time qPCR system (Applied Biosystems, Norwalk, CT, USA, #4407205). Stem-looped qPCR was used to measure the overexpression of hsa-miR-1-3p. Primers used for PCR are listed in the Supplementary Material (see Table S1).

### 2.7. miRNA Sequence Alignment

MicroRNA sequences were obtained from miRbase, while LOC730101 and all other mRNA sequences were directly downloaded from the NCBI. These sequences were then subjected to alignment analysis using MEGA v11.0 [19].

### 2.8. Gene Expression Analysis of TCGA and Existing GEO Datasets

The TCGA_PRAD and WCDT_mCRPC datasets were accessed using TCGAbiolinks v2.32.0. Gene expression datasets related to enzalutamide resistance were retrieved from the Gene Expression Omnibus (GEO) database, specifically GSE136130 and GSE104935. FPKM values from GSE136130 and microarray data from GSE104935 for LOC730101 expression were examined. Transcriptome profiling was conducted with the R package SummarizedExperiments v1.34.0, and FPKM normalization was applied for this clinical data analysis [20].

### 2.9. Hi-C and Analysis

Hi-C libraries were prepared using the Arima Genomics Hi-C kit and protocol (Arima Genomics, Carlsbad, CA, USA). Sequencing was conducted by NovoGene Novaseq, generating 150 pair-end reads. Subsequently, the Hi-C data were processed through the Juicebox Hi-C pipeline, and differential loop calling was performed using Mustache-Diff v1.0.1 [21,22]. Hi-C data were normalized by Genome-Wide Knight–Ruiz (GW-KR) normalization and presented in WashU epigenome browser.

### 2.10. Statistical Analysis

Data in this study were analyzed using Prism 8.0 (GraphPad, San Diego, CA, USA). The sample size was set to a minimum of three independent experiments (biological replicates) and experimental findings were reliably reproducible. The statistical significance of multiple samples was determined by one-way ANOVA, and between two groups it was determined by a non-paired Student’s *t*-test. The N numbers of biological replicates are indicated in the figure legends. The error bars are labeled as standard deviations. Differences were considered statistically significant at *p* < 0.05. *p* values are individually marked on the graph. “NS” indicates not significant when *p* > 0.05.

## 3. Results

### 3.1. The lncRNA LOC730101 Is Highly Expressed in Darolutamide-Resistant Cells

**Figure 1:**
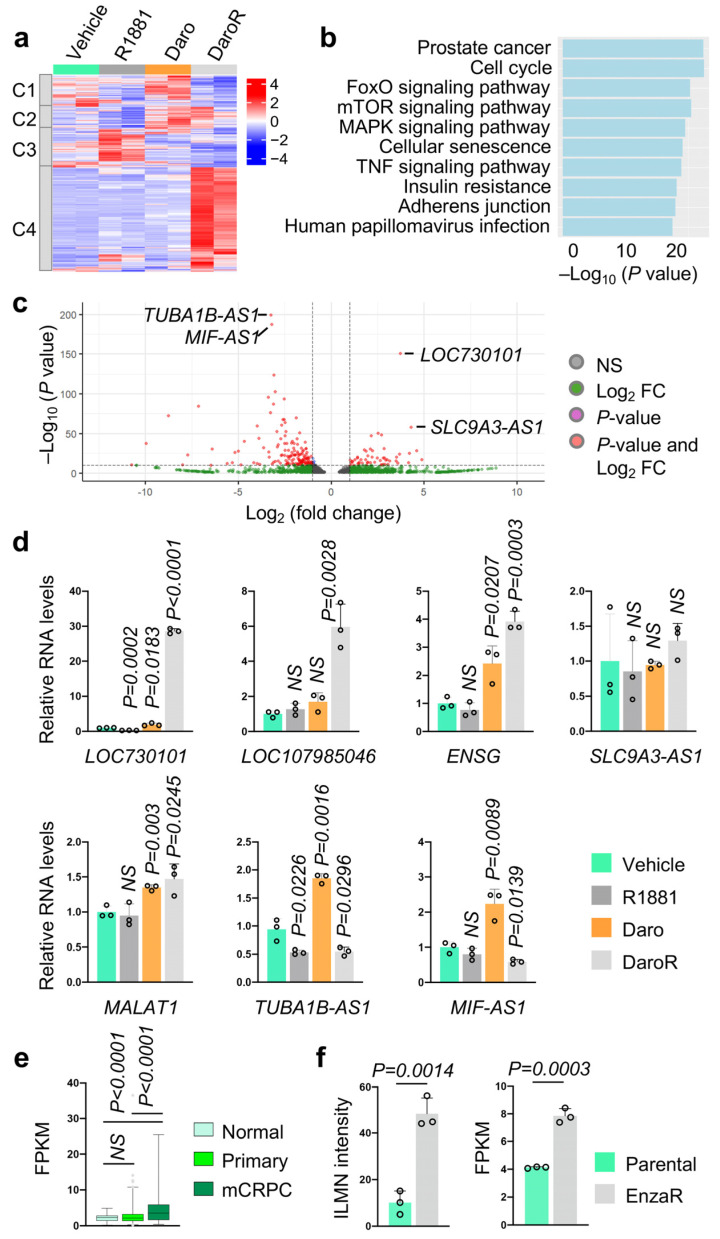
Identification of differentially expressed lncRNAs in darolutamide-resistant prostate cancer cells. (**a**) The androgen-regulated lncRNA transcriptome of prostate cancer LNCaP cells. The heatmap represents 1714 lncRNAs that are differentially expressed in LNCaP cells in response to 24 h treatment of Vehicle (DMSO), R1881 (10 nM), darolutamide (10 µM)/Daro, and darolutamide-resistant cells/DaroR. (**b**) The chart presents the top enriched pathways of differentially expressed lncRNA genes in DaroR cells, identified using NCPATH KEGG enrichment analysis. (**c**) Volcano plot shows the most differentially expressed lncRNAs with *p*-value and log2FoldChange (Log2FC). (**d**) Validation of expression of differentially expressed lncRNAs by RT-qPCR. (**e**) Expression of LOC730101 in the normal (N = 52), primary tumor (N = 501), and metastatic CRPC (mCRPC) tissues (N = 99) in TCGA_PRAD and WCDT_mCRPC datasets. (**f**) Expression of LOC730101 in enzalutamide-resistant (EnzaR) C4-2B cells (GEO access numbers: GSE104935 and GSE136130). All individual listed *p* values are in comparison with the first control column. NS, not significant.

We established darolutamide-resistant prostate cancer LNCaP cells by treating LNCaP cells with increasing concentrations of darolutamide until reaching 10 μM, and cells were maintained in culture media containing 10 μM darolutamide for 12 months to ensure that the resistance was fully established. The LNCaP cell line is the most widely used AR-positive prostate cancer cell line [23]. It contains the AR T877A mutation, yet its AR transcriptional activity remains intact. To identify lncRNAs specifically expressed in darolutamide-resistant prostate cancer cells, we performed RNA sequencing on darolutamide-resistant cells (DaroR), parental LNCaP cells treated with DMSO (Vehicle), LNCaP cells treated with 10 nM synthetic AR agonist R1881 (R1881), and LNCaP cells transiently treated with 10 μM darolutamide (Daro) for 24 h. This analysis revealed 1714 differentially expressed lncRNAs in DaroR cells compared to the other three treatment samples, with 1084 upregulated and 630 downregulated genes. Figure 1a shows the clustering of these 1714 differentially expressed genes. KEGG pathway analysis indicates that the cell cycle and prostate cancer pathways are the most significantly associated with these differentially expressed lncRNAs (Figure 1b). The volcano plot highlighted TUBA1B-AS1 and MIF-AS1 as the most significantly downregulated, and LOC730101 and SLC9A3-AS1 as the most significantly upregulated lncRNAs in DaroR cells (Figure 1c).

We subsequently conducted RT-qPCR to validate the expression levels of the identified lncRNAs in these four samples. In general, the RT-qPCR results largely mirrored the RNA sequencing trends. In particular, LOC730101 was notably distinguished by its substantially high expression in DaroR cells, suggesting its significance as a key lncRNA linked to darolutamide resistance (Figure 1d). In contrast, SLC9A3-AS1 exhibited overall low expression across all the samples. Furthermore, LOC730101 demonstrated a unique antiandrogen response; it was suppressed by R1881 treatment yet markedly upregulated in response to darolutamide, showing a nearly 30-fold increase in expression in DaroR cells.

LOC730101 is significantly overexpressed in the malignant tissues of non-small cell lung cancer (NSCLC) and osteosarcoma, where it plays a critical role in promoting cell proliferation and survival, respectively [24,25]. In NSCLC, LOC730101 activates the Wnt/β-catenin signaling pathway, while in osteosarcoma, it triggers the AMPK pathway, particularly under conditions of energy stress [26,27]. However, the role of LOC730101 in prostate cancer has not been explored. Therefore, we investigated whether LOC730101 expression is altered in prostate tumors, particularly in advanced stages such as metastatic CRPC. We performed transcriptomic analysis on the TCGA_PRAD and WCDT_mCRPC datasets to determine LOC730101 levels. As shown in Figure 1e, LOC730101 levels are not differentially expressed in primary prostate tumors but are significantly elevated in metastatic CRPCs, indicating that our cellular discovery mirrors clinical outcomes. To assess if other clinically used second-generation antiandrogens, such as enzalutamide, can increase LOC730101 expression, we analyzed two previously published GEO datasets with transcriptome results from enzalutamide-resistant C4-2B cells, an established CRPC cell line (GEO access numbers GSE104935 and GSE136130) [28,29]. In both studies, LOC730101 is elevated in enzalutamide-resistant cells (Figure 1f), reinforcing that its expression might be closely linked to antiandrogen resistance.

### 3.2. Depleting LOC730101 Reduces the Growth of Darolutamide-Resistant Cells by Downregulating Cell Cycle Gene Expression

**Figure 2:**
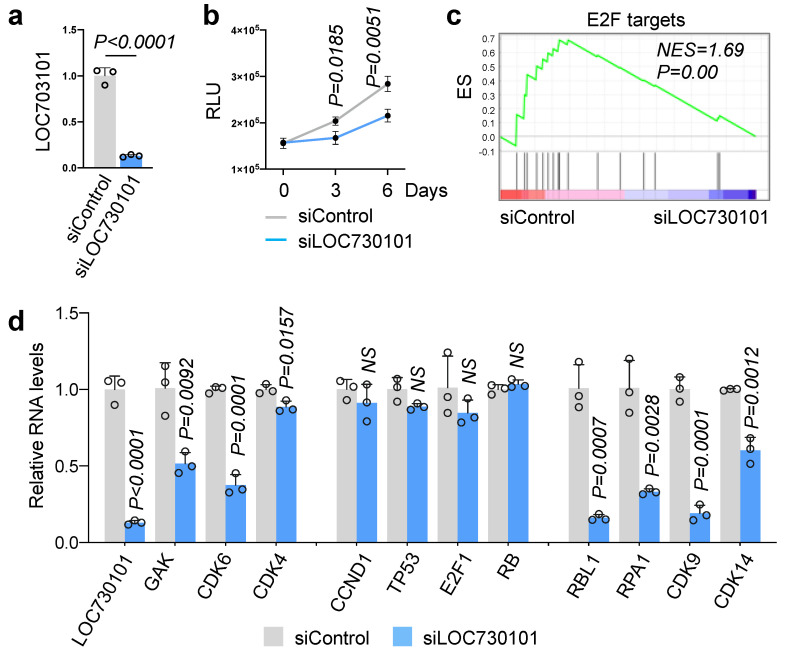
Impact of LOC730101 on gene expression in darolutamide-resistant (DaroR) cells. (**a**) Knockdown of LOC730101 by siRNA in DaroR cells. (**b**) Cell growth assay performed post-siRNA knockdown of LOC730101. Day 0 marks the time when siRNA knockdown was performed. N = 4. (**c**) GSEA analysis of differentially expressed genes in siLOC730101-DaroR. (**d**) Expression analysis of differentially expressed genes associated with the cell cycle and E2F pathways by RT-qPCR. NS, not significant.

Having established a link between LOC730101 expression and darolutamide resistance, we next explored the impact of this lncRNA on the survival of DaroR cells. Knocking down LOC730101 in DaroR cells using siRNA resulted in an over 80% reduction in its expression (Figure 2a) and significantly decreased cell growth (Figure 2b). DaroR cells were cultured in a medium containing 10 μM darolutamide. This result demonstrates that downregulating LOC730101 enhances sensitivity to darolutamide. Subsequent RNA sequencing analysis revealed changes in gene expression, with 1880 genes upregulated and 1463 downregulated in LOC730101-ablated DaroR cells (padj < 0.05). Gene Set Enrichment Analysis (GSEA) identified E2F target genes as the predominantly affected genes in LOC730101-ablated cells, suggesting a reduction in cell cycle progression (Figure 2c and Figure S1). This indicates that LOC730101 may promote cell growth by enhancing the E2F pathway.

We further determined the expression of key genes implicated in the E2F pathway (*CDK4*, *CDK6*, *GAK*, *CCND1*, *TP53*, *E2F1*, and *RB*), along with the significantly downregulated cell cycle-related genes identified by RNA-seq (*CDK9*, *CDK14*, *RBL1*, and *RPA1*). Figure 2d demonstrates significant changes in the expression of *CDK6*, *CDK9*, *CDK14*, *GAK*, *RBL1*, and *RPA1* due to siLOC730101 treatment. These results indicate that LOC730101 enhances the growth of darolutamide-resistant cells, and this effect is likely through regulating cell cycle-related genes associated with the E2F pathway. We further conducted cell cycle analysis and found that depletion of LOC730101 increased the percentage of apoptotic cells from 1.73% to 7.73% (Figure S2). This increase in apoptosis is accompanied by a slight decrease in G1 phase cells, potentially indicating G1 phase cells are undergoing apoptosis. The disruption of the cell cycle and the resulting stress may lead to the activation of apoptosis and a delay in DNA synthesis or replication.

### 3.3. LOC730101 Regulates miR-1-3p RNA Level

**Figure 3:**
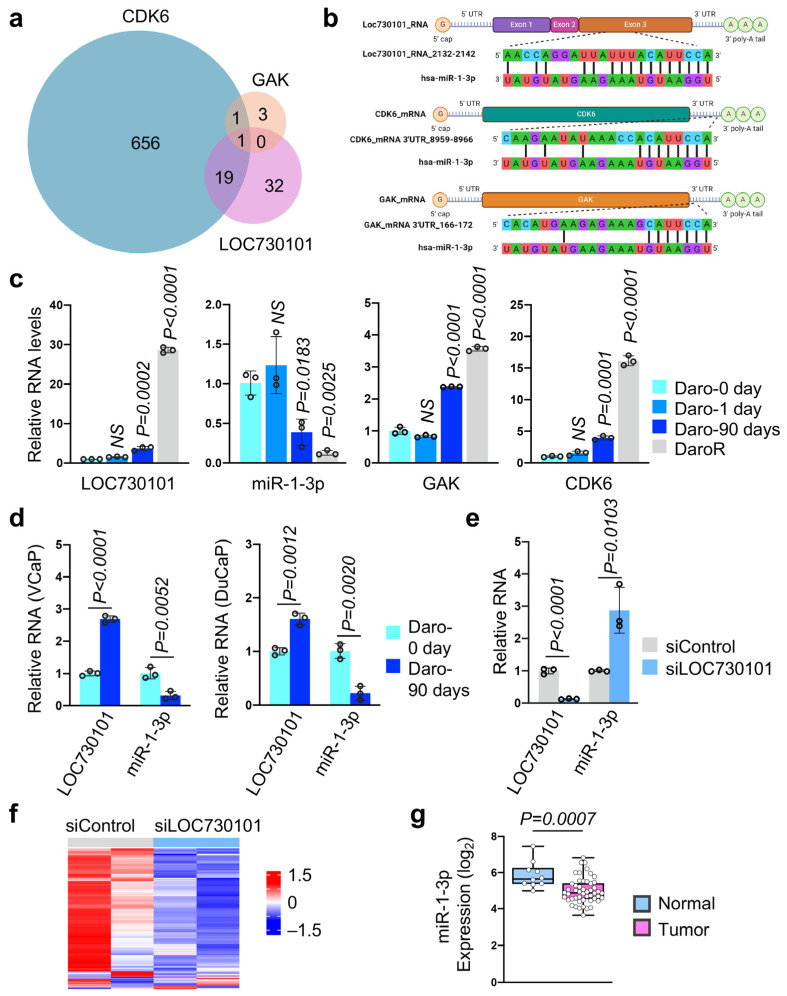
LOC730101 antagonizes hsa-miR-1-3p. (**a**) Venn diagram shows the overlap of LOC730101-, GAK-, and CDK6-associated microRNAs. (**b**) Sequence alignment of miR-1-3p with LOC730101, CDK6, and GAK. (**c**) Expression of LOC730101, miR-1-3p, GAK, and CDK6 throughout the darolutamide treatment course. NS, not significant. (**d**) Expression of LOC730101 and miR-1-3p in VCaP and DuCaP cells upon long-term treatment of darolutamide. (**e**) Knockdown of LOC730101 by siRNA enhances the expression of miR-1-3p. (**f**) Expression heatmap of miR-1-3p-targeted genes in DaroR cells with LOC730101 siRNA knockdown. A total of 852 miR-1-3p target genes were identified by TargetScan. (**g**) miR-1-3p expression was analyzed in normal benign tissue (N = 10) and prostate tumors (N = 50). The gene expression data were obtained from the GEO archive under accession number GSE45604 with microarray intensity measurements.

LncRNAs can act as miRNA sponges by binding to miRNAs through complementary sequences, thereby inhibiting miRNA activity and altering the stability of mRNAs targeted by these miRNAs [15]. To investigate whether LOC730101 regulates downstream events through this molecular mechanism, we searched for potential miRNA candidates using the Tarbase v7.0 and LncBase v2.0 databases. Interestingly, microRNA miR-1-3p was identified as a common miRNA targeting GAK, CDK6, and LOC730101 (Figure 3a). Sequence alignment confirmed complementary matching between miR-1-3p and the sequences of LOC730101, GAK, and CDK6 (Figure 3b). Additionally, miR-1-3p also exhibits significant sequence complementarity with CDK9 and CDK14, two differentially expressed genes identified by RNA-seq (Figure S3).

miR-1-3p is considered a tumor-suppressive microRNA encoded by the miR-1-1 and miR-1-2 genes on chromosomes 20q13.33 and 18q11.2, respectively. miR-1-3p is frequently downregulated in various tumors, including prostate cancer [30,31]. In order to further investigate the relationship between LOC730101 and miR-1-3p, we then analyzed the miR-1-3p expression level and its potential target genes *GAK* and *CDK6* in LNCaP cells treated with darolutamide for varying durations, including 24 h and 90 days, and in resistant DaroR cells. An inverse relationship was observed in DaroR cells between the RNA levels of miR-1-3p and LOC730101, GAK, and CDK6 (Figure 3c). We also investigated whether this observation extends to other AR-positive cell lines. Both VCaP and DuCaP cells express high levels of wild-type AR and are hormone-sensitive [23]. Therefore, we have included these two AR-positive cell lines in our study. Similarly, after 90 days of darolutamide treatment, both VCaP and DuCaP prostate cancer cells exhibited upregulation of LOC730101 and downregulation of miR-1-3p (Figure 3d). Additionally, we measured the expression levels of potential miR-1-3p target genes, including *GAK*, *CDK6*, *CDK9*, and *CDK14*, and observed varying degrees of upregulation in these genes (Figure S4).

In DaroR cells with siRNA LOC730101 knockdown, miR-1-3p levels were significantly elevated, indicating that LOC730101 exerts a negative regulatory effect on miR-1-3p (Figure 3e). To further elucidate the impact of LOC730101 on miR-1-3p-regulated pathways, we conducted an unbiased analysis using RNA-seq data from siRNA LOC730101 knockdown cells. TargetScan v7.0 identified 852 genes as potential miR-1-3p targets annotated in our RNAseq analysis (Figure 3f). The heatmap shows that 89.3% (761/852) of these miR-1-3p target genes were downregulated in siRNA LOC730101 knockdown DaroR cells, reinforcing the conclusion that LOC730101 negatively regulates miR-1-3p, thereby modulating the expression of miR-1-3p target genes.

To explore the clinical relevance of this observation, we further analyzed miR-1-3p levels in clinical samples from the GSE45604 dataset [32] and found significantly lower miR-1-3p levels in prostate tumor samples compared to normal benign tissue (Figure 3g). Further categorizing the prostate tumor samples by different Gleason scores revealed that the expression levels of miR-1-3p are lower in tumors with Gleason scores of 6 and 7, as well as in those with scores above 7 (Figure S5). Collectively, these findings indicate that LOC730101 acts as a competitor to miR-1-3p, and its ablation could enhance the activity of miR-1-3p, thereby affecting cell proliferation.

### 3.4. miR-1-3p Overexpression Suppresses Prostate Cancer Cell Proliferation

**Figure 4:**
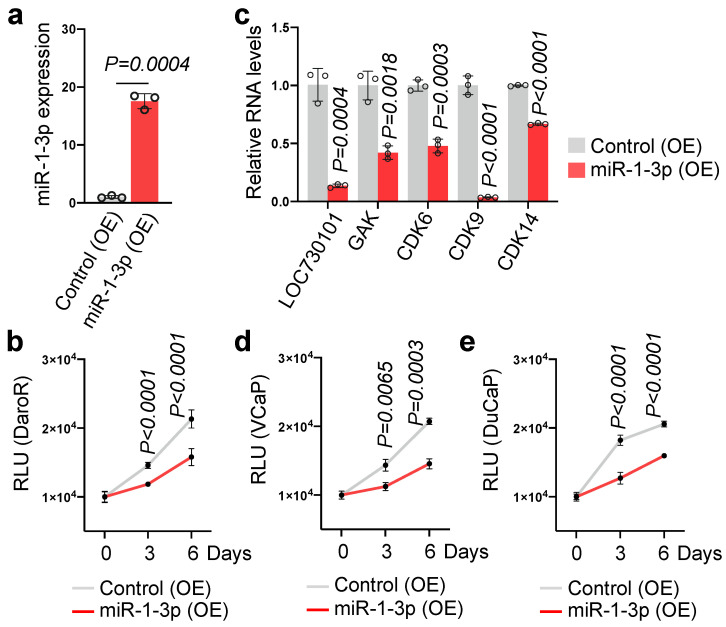
Exogenous expression of miR-1-3p inhibits prostate cancer cell growth. (**a**) Overexpression of miR-1-3p in DaroR cells by lentiviral transfection. The expression level of miR-1-3p was determined by stem-loop RT-qPCR. (**b**) Cell growth assay was conducted on DaroR cells with stable expression of miR-1-3p. (**c**) mRNA levels of LOC730101, GAK, CDK6, CDK9, and CDK14 in DaroR cells overexpressing miR-1-3p were determined by RT-qPCR. (**d**) Cell growth assay of VCaP cells with stable expression of miR-1-3p. (**e**) Cell growth assay of DuCaP cells with stable expression of miR-1-3p. OE, overexpression.

To verify the anti-proliferative effect of miR-1-3p in DaroR cells, we successfully introduced exogenous miR-1-3p into these cells using a lentiviral delivery system, as shown in Figure 4a. The overexpression of miR-1-3p led to a significant reduction in cell growth, indicating that upregulating miR-1-3p enhances sensitivity to darolutamide (Figure 4b). This growth inhibition was accompanied by decreased expression of miR-1-3p target genes, including *GAK*, *CDK6*, and *CDK14*, as confirmed by RT-qPCR analysis (Figure 4c). Interestingly, a concurrent reduction in LOC730101 expression was also observed, suggesting a potential negative feedback mechanism regulating LOC730101 (Figure 4c).

Furthermore, to confirm the tumor-suppressive role of miR-1-3p, we examined its effect on other prostate cancer cell lines, VCaP and DuCaP. Lentiviral vector-mediated overexpression of miR-1-3p in these cells similarly resulted in impaired cancer cell growth (Figure 4d,e) and downregulation of both LOC730101 and the cell cycle genes (Figure S6), indicating its broad tumor-suppressive function in prostate cancer. These results suggest miR-1-3p as a potential therapeutic molecule in targeting prostate cancer, particularly in overcoming resistance to antiandrogen therapies such as darolutamide.

### 3.5. LOC730101 Is Located in a Specific Genomic Locus Induced for Expression in DaroR Cells

**Figure 5:**
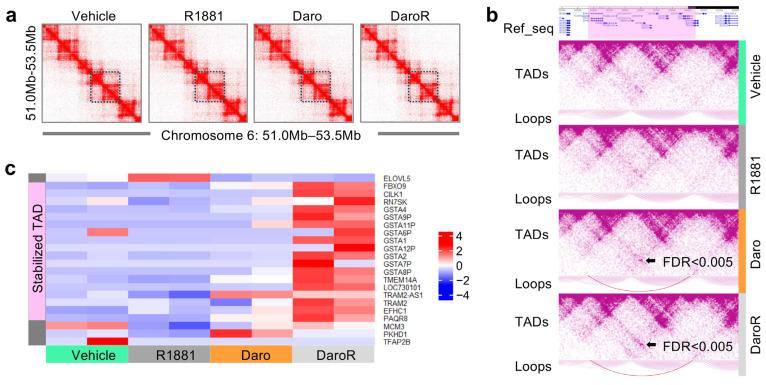
*LOC730101* is positioned in a genomic locus within a topologically associating domain (TAD) activated in darolutamide-resistant cells. (**a**) Hi-C analysis of the TAD arrangement at the *LOC730101* locus on chromosome 6 in Vehicle, R1881, Daro, and DaroR cells. The blue squares label the genomic region from chr6: 52,270,000 to 52,930,000. (**b**) Identification of unique differential loops established at the *LOC730101* genomic locus. The purple-highlighted region in Ref-seq corresponds to the stabilized TAD containing *LOC730101*. The differential loops are shown in dark purple and only identified in Daro and DaroR Hi-C. The black arrows mark the differential chromatin loops with FDR<0.005. (**c**) The heatmap displays the expression levels of genes within the stabilized TAD containing LOC730101, as determined by RNA sequencing.

To further elucidate the mechanisms underlying the upregulation of LOC730101 in DaroR cells, we examined its genomic context. LOC730101 is located on chromosome 6, near a cluster of GSTA genes and several other genes. Hi-C sequencing was performed on four samples: DaroR cells, parental LNCaP cells treated with DMSO (Vehicle), LNCaP cells treated with 10 nM R1881 (R1881), and LNCaP cells treated with 10 μM darolutamide (Daro) for 24 h. Hi-C analysis revealed that LOC730101 and these neighboring genes form two small topologically associating domains (TADs) encompassing approximately 660 kilobases, covering the genomic region from chr6: 52,270,000 to 52,930,000 (Figure 5a).

Although the overall TAD structures remained consistent across all four treatment samples, differential loop analysis using the Mustache tool identified a unique chromatin loop with enriched contact counts in Daro and DaroR cells, but not in Vehicle and R1881 cells (FDR < 0.005 in Figure 5b). This chromatin loop encompasses the two TADs containing the *LOC730101* gene and indicates the formation of a larger stabilized TAD. RNA sequencing results further demonstrate a significant increase in gene expression for all genes within this region in DaroR cells, but not for genes outside this region (Figure 5c).

This observation suggests that darolutamide treatment may induce changes in the local chromatin architecture, leading to increased expression of genes within this region in DaroR cells, ultimately contributing to drug resistance.

## 4. Discussion

**Figure 6:**
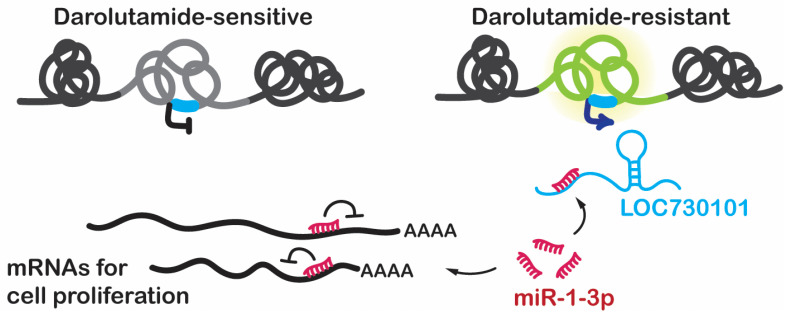
A proposed model of the LOC730101-miR-1-3p regulatory axis in darolutamide-resistant cells. The *LOC730101* gene locus is labeled in blue, and the activated TAD is highlighted in yellow.

In this study, we investigated the function of lncRNAs in darolutamide resistance in prostate cancer, and we identified LOC730101 as an important lncRNA in promoting the proliferation of prostate cancer cells that are resistant to darolutamide, the latest FDA-approved second-generation antiandrogen drug. Long-term darolutamide treatment significantly induces the overexpression of LOC730101, suggesting an adaptive cellular response counteracting the therapeutic effects of darolutamide (Figure 6).

Our further analyses identified miR-1-3p as a direct target of LOC730101. miR-1-3p is known for its tumor-suppressive properties in various cancer types. Consistent with previous studies, we found that miR-1-3p expression is downregulated in prostate tumors. Importantly, overexpression of miR-1-3p reduces the proliferation of darolutamide-resistant cells, indicating that its tumor-suppressive activity is maintained in resistant cells. We found that miR-1-3p targets and downregulates genes essential for cell cycle progression and proliferation, such as CDK6 and GAK, in the context of darolutamide resistance.

It has been reported that miR-1-3p can be silenced by several lncRNAs such as MALAT1, DANCR, and RMRP in cancers [33,34,35,36]. However, the regulation of miR-1-3p in prostate cancer with antiandrogen drug resistance remains unclear. Our study indicates that LOC730101 likely regulates miR-1-3p through a “sponge” mechanism, where it binds to miR-1-3p via complementary base pairing and sequesters it away from its target mRNAs. Although we have not demonstrated a direct interaction between miR-1-3p and LOC730101 in our study, a HITS-CLIP to profile direct miRNA-target interactions in human brain tissues reported such an interaction, which is documented on the DIANA Tools website [37], providing further support for our findings. In our working model, this interaction between LOC730101 and miR-1-3p leads to the upregulation of miR-1-3p target genes, promoting cell proliferation (Figure 6).

This study demonstrates that although the overall TAD structure remains stable, unique chromatin loops can form in response to darolutamide treatment, leading to increased expression levels of LOC730101. This suggests that gene regulation at this genomic locus is dynamic under darolutamide treatment, likely driven by localized chromatin interactions. The rearrangement of chromatin loops may influence the recruitment of transcription factors and coregulators or even alter chromatin accessibility. This study shows that the resulting upregulation of LOC730101 and potentially other critical genes within the TADs contribute to cancer cell survival and proliferation. Understanding these chromatin dynamics offers novel therapeutic targets that could be leveraged to mitigate resistance to darolutamide in prostate cancer.

## 5. Conclusions

This study demonstrates that the LOC730101/miR-1-3p axis plays a crucial role in darolutamide resistance in LNCaP cells, making it a potential target for overcoming antiandrogen resistance in advanced prostate cancer. Future research should extend these findings to additional cell lines, animal models, or clinical samples when available. Additionally, investigating the regulation of LOC730101 expression, its interactions with other molecular pathways, and the involved chromatin dynamics would be particularly valuable. The results from these studies could identify novel regulatory pathways to enhance the efficacy of antiandrogen therapy.

## Data Availability

The RNA sequencing and Hi-C data presented in these studies are available through GEO datasets with access numbers: GSE269644 and GSE269645.

## Supplementary Materials

The following supporting information can be downloaded at: https://www.mdpi.com/article/10.3390/cancers16142594/s1, Figure S1: GO and KEGG pathway analyses of siLOC730101 in darolutamide-resistant (DaroR) cells. Figure S2: Cell cycle analysis of DaroR cells with siLOC730101 knockdown. Figure S3: Sequence alignment of miR-1-3p with CDK9 and CDK14. Figure S4: Expression of LOC730101, GAK, CDK6, CDK9, and CDK14 in VCaP and DuCaP cells upon long-term treatment of darolutamide in VCaP cells and DuCaP cells. Figure S5: miR-1-3p expression was analyzed in clinical samples by different Gleason scores. Figure S6: Exogenous expression of miR-1-3p reduces the expression of its target genes in VCaP and DuCaP cells. Table S1: A list of primers that have been used in this study.
