# Supplementary material for: LncRNA LOC730101 Promotes Darolutamide Resistance in Prostate Cancer by Suppressing miR-1-3p
Source: Cancers (Basel). 2024 Jul 20;16(14):2594. doi: 10.3390/cancers16142594 (PMC11274508; doi:10.3390/cancers16142594)
Supplement: Supplementary file 1 [file cancers-16-02594-s001.zip › cancers-3076895-supplementary.pdf]

cancers

MDPI

Supplementary Figures

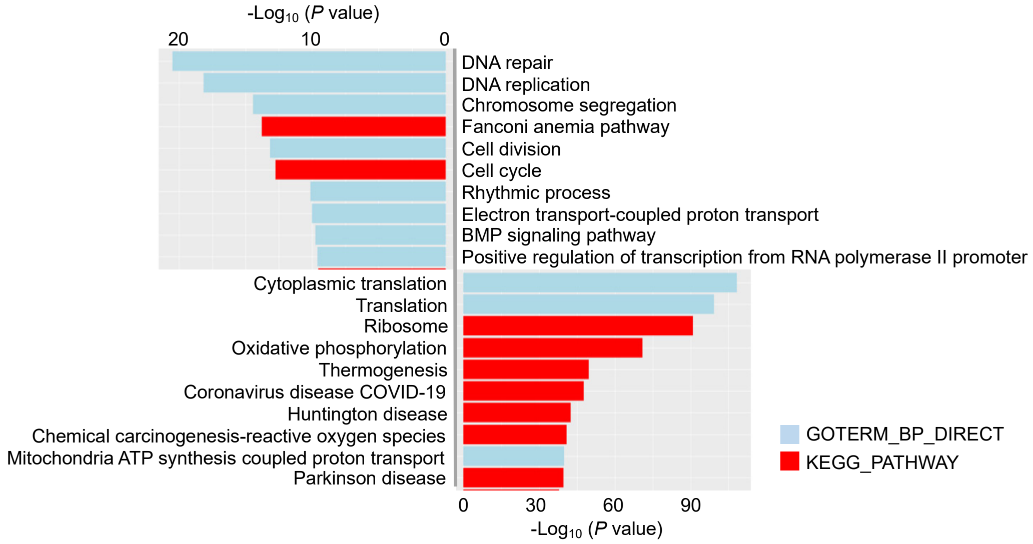

Figure S1. GO and KEGG pathway analyses of siLOC730101 in darolutamide-resistant (DaroR) cells.

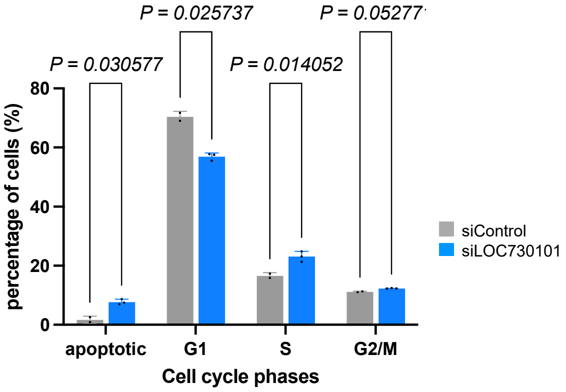

Figure S2. Cell cycle analysis of DaroR cells with siLOC730101 knockdown. Propidium iodide staining was performed and followed by FACS analysis.

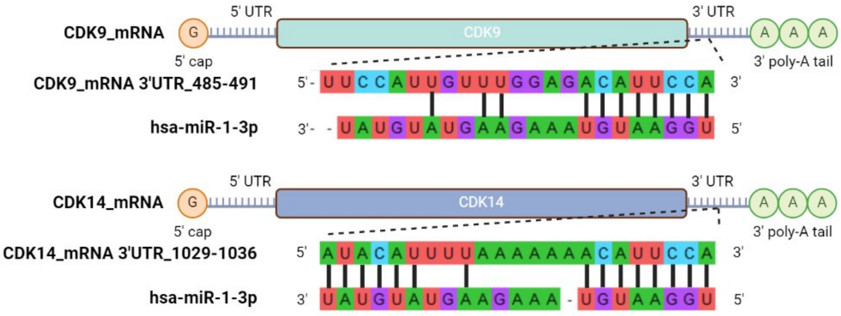

Figure S3. Sequence alignment of miR-1-3p with CDK9 and CDK14.

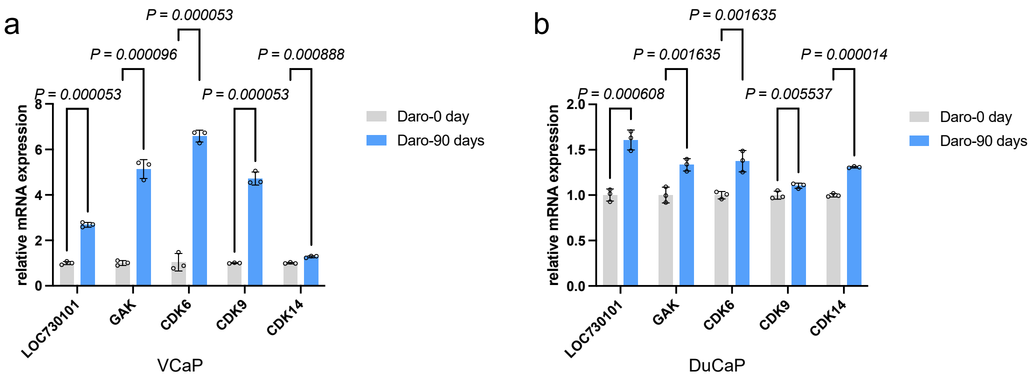

Figure S4. Expression of LOC730101, GAK, CDK6, CDK9, and CDK14 in VCaP and DuCaP cells upon long-term treatment of darolutamide in VCaP cells (a) and DuCaP cells (b).

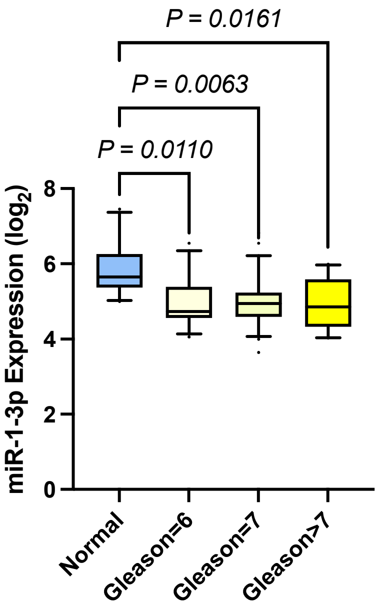

Figure S5. miR-1-3p expression was analyzed in normal benign tissue (N=10) and prostate tumors categorized by different Gleason scores (Gleason=6, N=15; Gleason=7, N=25; Gleason>7, N=10). The gene expression data were obtained from the GEO archive under accession number GSE45604 with the microarray intensity measurements.

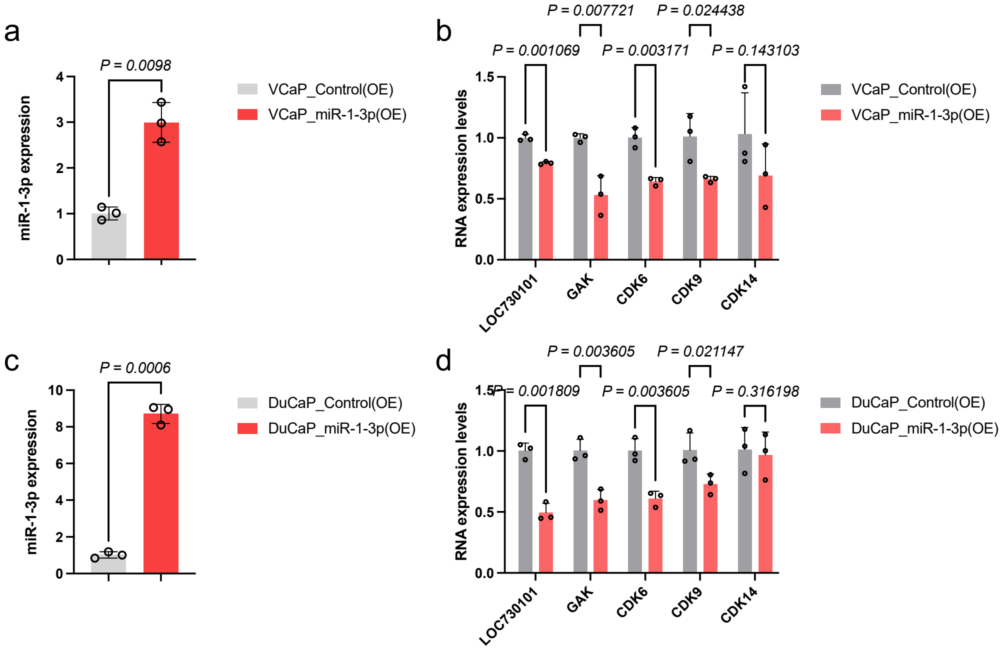

Figure S6. Exogenous expression of miR-1-3p reduces the expression of its target genes in VCaP and DuCaP cells. (a) Overexpression of miR-1-3p in VCaP cells. The miR-1-3p level was determined by qPCR. (b) mRNA levels of miR-1-3p target genes in VCaP cells determined by RT-qPCR. (c) Overexpression of miR-1-3p in DuCaP cells. (d) mRNA levels of miR-1-3p target genes in DuCaP cells determined by RT-qPCR.

Table S1. A list of primers that have been used in this study.

Primer names	Primer equences (5' to 3')
LOC730101-F	ACCAAGAGGGTTGACGTTTG
LOC730101-R	CTCAGTGGCTTTGGGAGTTC
ENSG00000273179-F	CTGGACCCAGCTGCTACATT
ENSG00000273179-R	GAGTTCCACCACCACAGGG
MIF-AS1-F	GAGACGAGATGTGGCTGGAG
MIF-AS1-R	GTTGCTCTCCTCCGAACCTG
SLC9A3-AS1-F	TCTTGTTCTGGTTGAGCCCC
SLC9A3-AS1-R	CTCACACCCAAGGTCTGTCC
TUBA1B-AS1-F	AAGACACCGACCAGGGAATG
TUBA1B-AS1-R	TAATGAGACGTCCAGGGGGA
MALAT1-F	TGCCTTGTGAGCACTTTCAG
MALAT1-R	ACGTGAAAACCCACTCTTGG
CDK6-F	GGATAAAGTTCCAGAGCCTGGAG
CDK6-R	GCGATGCACTACTCGGTGTGAA
CDK4-F	CCATCAGCACAGTTCGTGAGGT
CDK4-R	TCAGTTCGGGATGTGGCACAGA
GAK-F	TGCAAGGAGACGTGCTCATCGT
GAK-R	TTTCACAGTGGTGGCGTTCCGA
CDK14-F	CCATACCAAGGAGACGCTGACA
CDK14-R	AGACAGACCTCGCAGCAACTGA
CDK9-F	CCATTACAGCCTTGCGGGAGAT
CDK9-R	CAGCAAGGTCATGCTCGCAGAA
RB1-F	CAGAAGGTCTGCCAACACCAAC
RB1-R	TTGAGCACACGGTCGCTGTTAC
E2F1-F	GGACCTGGAAACTGACCATCAG
E2F1-R	CAGTGAGGTCTCATAGCGTGAC
RBL1-F	CGAACTGACAGTGGGAGTCTTC
RBL1-R	TCTCTTAGCACTCCCTGCGGTA
RPA1-F	CGAGTCTCTGATTTCGGTGGAC
RPA1-R	GGCTTGTCCTTCTGCGTCAAAC
CCND1-F	TCTACACCGACAACTCCATCCG
CCND1-R	TCTGGCATTTTGGAGAGGAAGTG
CCNE1-F	TGTGTCCTGGATGTTGACTGCC
CCNE1-R	CTCTATGTCGCACCACTGATACC
TP53-F	CCTCAGCATCTTATCCGAGTGG
TP53-R	TGGATGGTGGTACAGTCAGAGC
StemloopMir1	GTCGTATCCAGTGCAGGGTCCGAGGTATTCGCACTGGATACGACATACAT
F1-miR	CACGCATGGAATGTAAAG
R1-miR	CCAGTGCAGGGTCCGAGGTA
